# The protective effect of mushroom consumption on depressive symptoms in Korean population

**DOI:** 10.1038/s41598-022-26549-5

**Published:** 2022-12-19

**Authors:** Sung Keun Park, Chang-Mo Oh, Jae-Hong Ryoo, Ju Young Jung

**Affiliations:** 1 Center for Cohort Studies, Total Healthcare Center, Kangbuk Samsung Hospital, Sungkyunkwan University School of Medicine, Seoul, Republic of Korea; 2 Department of Preventive Medicine, School of Medicine, Kyung Hee University, Seoul, Korea; 3 Department of Occupational and Environmental Medicine, School of Medicine, Kyung Hee University, Seoul, Korea; 4 Total Healthcare Center, Kangbuk Samsung Hospital, Sungkyunkwan University, School of Medicine, 67, Sejong-daero, Jung-gu, Seoul, 04514 Korea

**Keywords:** Medical research, Risk factors

## Abstract

Mushrooms are nutraceutical food with health benefit. However, available data is still limited in identifying the effect of mushrooms consumption on depressive symptoms. In a cohort of 87,822 Korean, we longitudinally assessed the risk of depressive symptoms according to mushrooms consumption. Study participants were categorized into 5 groups by the frequency of one serving size of mushrooms (30 g) as follows: rare/never, < 1/month, 1/month–1/week, 1–3/week, ≥ 3/week. The development of depressive symptoms was determined in Center for epidemiological studies-depression scale ≥ 16. Cox proportional hazards model was used to calculate adjusted hazard ratio (HR) and 95% confidence intervals (CI) for depressive symptoms (adjusted HR [95% CI]). Subgroup analysis was performed for gender and age. Compared with group with rare/never consumption, groups with mushrooms consumption ≥ one serving size/month had the significantly decreased levels in adjusted HR and 95% CI for depressive symptoms (rare/never consumption: reference, < 1/month: 0.92 [0.83–1.02], 1/month–1/week: 0.88 [0.83–0.94], 1–3/week: 0.88 [0.82–0.94], ≥ 3/week: 0.86 [0.80–0.93]). This association was similarly observed in both gender and age subgroup analyses. However, women and participants ≥ age of 40 showed the more prominent association than men and participants < age of 40.

## Introduction

Depression is a serious neuropsychiatric disorder, leading to depressed mood, loss of interest, low self-esteem, cognitive impairment, sleep disturbance and eating disorder^1^. Studies have demonstrated that depression is associated with the development and progression of chronic illness including diabetes mellitus (DM), hypertension and cardiovascular disease^2–4^. Lifetime prevalence of depression varies among countries from 16.2% in USA to 53% in low income countries^5,6^. World Health Organization (WHO) has reported that more than 300 million people suffer from depressive symptoms over all ages globally^7^. Therefore, it is important health challenge to investigate ways to alleviate depressive symptoms.

It is widely believed that good dietary habit and nutritional foods are helpful in controlling mood disorder. In practice, a randomized trial indicated that improvement of diet is potentially effective in treating depression^8^. A meta-analysis from 21 studies of ten countries presented that the risk of depression was inversely associated with high intake of fruits, vegetables, whole grains, and antioxidants^9^.

Mushrooms are functional foods with high nutritional values and great sources for novel therapeutic compounds, linking to good dietary habit^10–12^ Studies have demonstrated that some types of mushrooms have therapeutic potential for cognitive impairments^13^ and Alzheimer’s disease^14^. Mushrooms are rich in bioactive components including antioxidants and neurotrophic factors, which are helpful in preventing neuropsychiatric disorders^15,16^. Several studies have suggested that mushrooms intake is potentially effective in attenuating depressive symptoms^17–19^. However, data is still insufficient to identify the association between association between comprehensive mushrooms intake and the risk of depressive symptoms.

Therefore, to identify the effect of mushrooms intake on depression, we longitudinally investigated the risk of depressive symptoms according to the consumption of mushrooms in 87,822 Korean adults.

## Results

The baseline clinical and sociodemographic characteristics among study groups are presented in Table 1. The cohort of present study is characterized by relatively young age with mean age of 39.6 ± 6.9 years and the preponderance of men with 56,112 male participants (63.89%). The age of study participants ranged from 18 to 87 years, and only 1.1% (n = 952) of the subjects were over 60 years of age.

**Table 1:** Baseline clinical characteristics of study participants according to the mushroom consumption.

Characteristics	Serving					P value
	Rare/never	< 1/month	≥ 1/month and < 1/week	≥ 1 and < 3/week	≥ 3/week	
Number	7665	3782	38,491	23,842	14,042	
Men	5790 (10.3%)	2565 (4.5%)	26,130 (46.5%)	14,511 (25.8%)	7116 (12.6%)	
Women	1875 (5.9%)	1217 (3.8%)	12,361 (38.9%)	9331 (29.4%)	6926 (21.8%)	
Age (year)	39.4 ± 7.3	39.5 ± 7.3	39.6 ± 6.9	39.5 ± 6.7	39.7 ± 6.7	0.026
Age ≥ 40 years (%)	42.6%	42.6%	43.8%	42.7%	44.4%	0.003
BMI (kg/m^2^)	23.9 ± 3.2	23.2 ± 3.1	23.4 ± 3.2	23.4 ± 3.2	23.2 ± 3.3	< 0.001
Average alcohol use (g/day)	18.0 ± 24.4	15.4 ± 20.3	15.6 ± 21.9	14.6 ± 21.9	13.5 ± 21.5	< 0.001
Total calorie intake (kcal/day)	1437.7 ± 627.5	1422.3 ± 541.6	1546.3 ± 586.8	1696.9 ± 621.7	1902.7 ± 870.8	< 0.001
Current smoker (%)	31.0%	25.5%	25.0%	23.0%	19.2%	< 0.001
Hypertension (%)	13.5%	10.5%	10.9%	10.8%	10.0%	< 0.001
DM (%)	5.0%	3.7%	3.7%	3.5%	3.7%	< 0.001
Married (%)	81.9%	85.0%	87.2%	89.8%	91.6%	< 0.001
High education (%)	71.0%	72.2%	74.9%	75.0%	71.9%	< 0.001
**Mushroom consumption (g/day)**	0 ± 0	0.5 ± 0.0	2.1 ± 0.9	6.5 ± 1.8	22.5 ± 16.1	< 0.001
Oyster mushroom	0 ± 0	0.3 ± 0.2	0.9 ± 0.7	3.1 ± 1.7	10.9 ± 9.2	< 0.001
Other mushroom	0 ± 0	0.2 ± 0.2	1.2 ± 0.9	3.4 ± 1.8	11.6 ± 9.4	< 0.001
Baseline CESD score	5.3 ± 4.1	5.0 ± 4.1	5.0 ± 4.1	4.9 ± 4.2	5.1 ± 4.2	0.014
Incident depressive symptom (n, [%])	1201 (15.7%)	551 (14.6%)	5522 (14.3%)	3516 (14.7%)	2135 (15.2%)	0.014

Continuous variables are expressed as mean (± SD), and categorical variables are expressed as number (percentage (%)).

*< 1/month *< 1 serving size/month, *1/month–1/week* 1 serving size/month ≤ and < 1 serving size/week, *1–3/week* 1 serving size/week ≤ and < 3 serving sizes/week, *≥ 3/week *≥ 3 serving sizes/week.

*BMI* body mass index, *DM* diabetes mellitus, *CESD* Center for Epidemiologic Studies Depression.

The proportion of participants consuming mushrooms ≥ one serving size/week was higher in women (51.2%) than men (38.4%). Consumption of oyster mushroom and other mushrooms was almost similar in each consumption group. Groups with mushrooms consumption > one serving size/month tended to have the higher levels in total calorie intake, the proportion of marriage and the proportion of high education, compared with rare/never consumption. In addition, they had the lower levels in the proportion of current smoker, average alcohol use, the prevalence of hypertension, baseline Center for epidemiological studies-depression (CES-D) score and the incidence of depressive symptoms than group with rare/never consumption.

During 5.8 years of median follow-up period, 12,925 subjects (14.3%) reported new onset of depressive symptoms (CES-D ≥ 16), and group with rare/never mushrooms consumption presented the highest values in the baseline CES-D score (5.3 ± 4.1) and the incidence of depressive symptoms (15.7%).

Table 2 shows the unadjusted and the multivariable adjusted HR and 95% CI for depressive symptoms according to the groups with mushrooms consumption. In all participants, compared with group with rare/never consumption, groups with mushrooms consumption ≥ one serving size/month had the significantly decreased levels in adjusted HR and 95% CI for depressive symptoms (rare/never consumption: reference, < 1/month: 0.92 [0.83–1.02], 1/month–1/week: 0.88 [0.83–0.94], 1–3/week: 0.88 [0.82–0.94], ≥ 3/week: 0.86 [0.80–0.93], P for trend < 0.001). Gender subgroup analysis indicated the similar pattern of relationship between mushrooms consumption and depressive symptoms in both men and women. However, women showed the more distinct association than men (rare/never consumption: reference, < 1/month: 0.82 [0.69–0.98], 1/month–1/week: 0.86 [0.77–0.96], 1–3/week: 0.85 [0.76–0.95], ≥ 3/week: 0.81 [0.72–0.92], P for trend < 0.001).

**Table 2:** Hazard ratio (HR) and 95% confidence intervals (CI) for depressive symptom (CESD ≥ 16) according to the mushroom consumption.

Characteristics	Serving					P for trend
	Rare/never	< 1/month	1/month–1/week	1–3/week	≥ 3/week	
**All participants (n)**	7665	3782	38,491	23,842	14,042	
Unadjusted HR	1.00 (Reference)	0.92 (0.84–1.02)	0.89 (0.84–0.95)	0.92 (0.86–0.98)	0.95 (0.89–1.02)	0.640
Adjusted HR	1.00 (Reference)	0.92 (0.83–1.02)	0.88 (0.83–0.94)	0.88 (0.82–0.94)	0.86 (0.80–0.93)	< 0.001
Incidence case [n, (%)]	1201 (15.7%)	551 (1.46%)	5522 (14.3%)	3516 (14.7%)	2135 (15.2%)	
Incidence density	31.4	29.0	28.1	29.0	30.0	
Person year	38,291	18,984	196,448	121,386	71,172	
**Men (n)**	5790	2565	26,130	14,511	7116	
Unadjusted HR	1.00 (Reference)	0.96 (0.85–1.09)	0.88 (0.82–0.95)	0.90 (0.83–0.98)	0.94 (0.86–1.03)	0.178
Adjusted HR	1.00 (Reference)	0.97 (0.86–1.10)	0.89 (0.82–0.96)	0.89 (0.82–0.97)	0.90 (0.82–0.99)	0.021
Incidence case [n, (%)]	827 (14.3%)	353 (13.8%)	3389 (13.0%)	1911 (13.2%)	977 (13.7%)	
Incidence density	28.2	27.1	25.0	25.5	26.7	
Person year	29,345	13,043	135,639	75,036	36,6327	
**Women (n)**	1875	1217	12,361	9331	6926	
Unadjusted HR	1.00 (Reference)	0.79 (0.67–0.94)	0.83 (0.75–0.93)	0.82 (0.73–0.92)	0.79 (0.71–0.89)	0.002
Adjusted HR	1.00 (Reference)	0.82 (0.69–0.98)	0.86 (0.77–0.96)	0.85 (0.76–0.95)	0.81 (0.72–0.92)	0.005
Incidence case [n, (%)]	374 (19.9%)	198 (16.3%)	2133 (17.3%)	1605 (17.2%)	1158 (16.7%)	
Incidence density	41.8	33.3	35.1	34.6	33.5	
Person year	8945	5940	60,809	46,349	34,545	

Adjusting covariates: age, BMI, sex, alcohol intake, hypertension, diabetes, smoking, marital status, education, and total calorie intake (sex excluded in gender subgroup analysis).

*< 1/month *< one serving size/month, *1/month–1/week* one serving size/month ≤  ~  < one serving size/week, *1–3/week* one serving size/week ≤  ~  < three serving sizes/week, *≥ 3/week *≥ three serving sizes/week.

*Adjusted HR* multivariate-adjusted hazard ratio.

In age subgroup analysis by age of 40 years, subgroup with age < 40 years presented the marginally significant association between mushrooms consumption ≥ one serving size/week and the decreased risk of depressive symptoms (Table 3). However, in subgroup with age ≥ 40 years, participants consuming mushrooms ≥ one serving size/month had the significantly decreased HR and 95% CI for depressive symptoms (rare/never consumption: reference, < 1/month: 0.96 [0.82–1.12], 1/month–1/week: 0.80 [0.73–0.89], 1–3/week: 0.82 [0.74–0.91], ≥ 3/week: 0.79 [0.70–0.88], P for trend < 0.001).

**Table 3:** Hazard ratio (HR) and 95% confidence intervals (CI) for depressive symptom (CESD ≥ 16) according to the mushroom consumption in subgroups stratified by age.

Characteristics	Serving					P for trend
	Rare/never	< 1/month	1/month–1/week	1–3/week	≥ 3/week	
**Age < 40 years old**	4403	2171	21,621	13,666	7814	
Unadjusted HR	1.00 (Reference)	0.89 (0.78–1.02)	0.95 (0.87–1.03)	0.95 (0.87–1.03)	1.00 (0.91–1.09)	0.660
Adjusted HR	1.00 (Reference)	0.89 (0.78–1.02)	0.94 (0.86–1.02)	0.92 (0.84–1.00)	0.91 (0.83–1.00)	0.082
Incidence case [n, (%)]	692 (15.7%)	309 (14.2%)	3272 (15.1%)	2081 (15.2%)	1242 (15.9%)	
Incidence density	30.2	27.0	28.7	28.9	30.3	
Person year	22,909	11,432	113,955	72,068	41,048	
**Age ≥ 40 years old**	3262	1611	16,870	10,176	6228	
Unadjusted HR	1.00 (Reference)	0.97 (0.83–1.13)	0.82 (0.75–0.91)	0.88 (0.79–0.97)	0.89 (0.80–0.996)	0.214
Adjusted HR	1.00 (Reference)	0.96 (0.82–1.12)	0.80 (0.73–0.89)	0.82 (0.74–0.91)	0.79 (0.70–0.88)	< 0.001
Incidence case [n, (%)]	509 (15.6%)	242 (15.0%)	2250 (13.3%)	1435 (14.1%)	893 (14.3%)	
Incidence density	33.1	32.0	27.3	29.1	29.6	
Person year	15,382	7552	82,493	49,318	30,124	

Adjusting covariates: age, BMI, sex, alcohol intake, hypertension, diabetes, smoking, marital status, education, and total calorie intake.

*< 1/month *< one serving size/month, *1/month–1/week* one serving size/month ≤  ~  < one serving size/week, *1–3/week* one serving size/week ≤  ~  < three serving sizes/week, *≥ 3/week *≥ three serving sizes/week.

*Adjusted HR* multivariate-adjusted hazard ratio.

Table 4 shows the logistic regression analysis for the association between mushrooms consumption and depressive symptoms defined by multiple cutoffs of CES-D^15,16,20,22^. Groups with mushrooms consumption ≥ 1/month had lower adjusted odd ratio (OR) and 95% confidence interval for depressive symptoms defined by all cutoffs of CES-D^15,16,20,22^, compared with group with rare/never consumption.

**Table 4:** Odds ratio (OR) and 95% confidence intervals (CI) for depressive symptom (CESD ≥ 16, 20, 22, 25) according to the baseline mushroom consumption.

CESD cutoff point	Serving					P for trend
	Rare/never	< 1/month	1/month–1/week	1–3/week	≥ 3/week	
Number	10,406	4998	50,188	30,855	18,655	
**CESD ≥ 16**						
Unadjusted OR	1.00 (Reference)	0.88 (0.79–0.98)	0.81 (0.76–0.86)	0.81 (0.76–0.87)	0.91 (0.84–0.98)	0.037
Adjusted OR	1.00 (Reference)	0.87 (0.78–0.97)	0.80 (0.75–0.85)	0.75 (0.70–0.81)	0.75 (0.70–0.81)	< 0.001
Incidence case [n, (%)]	1316 (12.6%)	566 (11.3%)	5254 (10.5%)	3246 (10.5%)	2164 (11.6%)	
**CESD ≥ 20**						
Unadjusted OR	1.00 (Reference)	0.89 (0.78–1.02)	0.81 (0.75–0.88)	0.78 (0.71–0.85)	0.91 (0.83–0.99)	0.020
Adjusted OR	1.00 (Reference)	0.88 (0.77–1.00)	0.80 (0.74–0.87)	0.71 (0.65–0.78)	0.74 (0.67–0.81)	< 0.001
Incidence case [n, (%)]	810 (7.8%)	350 (7.0%)	3214 (6.4%)	1901 (6.2%)	1326 (7.1%)	
**CESD ≥ 22**						
Unadjusted OR	1.00 (Reference)	0.84 (0.72–0.97)	0.79 (0.72–0.86)	0.76 (0.69–0.83)	0.90 (0.82–1.00)	0.053
Adjusted OR	1.00 (Reference)	0.82 (0.71–0.95)	0.77 (0.71–0.85)	0.69 (0.62–0.76)	0.73 (0.65–0.81)	< 0.001
Incidence case [n, (%)]	645 (6.2%)	262 (5.2%)	2480 (4.9%)	1467 (4.8%)	1050 (5.6%)	
**CESD ≥ 25**						
Unadjusted OR	1.00 (Reference)	0.85 (0.71–1.01)	0.75 (0.68–0.84)	0.73 (0.65–0.81)	0.87 (0.77–0.98)	0.024
Adjusted OR	1.00 (Reference)	0.83 (0.69–0.98)	0.74 (0.66–0.82)	0.66 (0.58–0.74)	0.69 (0.61–0.78)	< 0.001
Incidence case [n, (%)]	455 (4.4%)	186 (3.7%)	1671 (3.3%)	991 (3.2%)	715 (3.8%)	

Adjusting covariates: age, BMI, sex, alcohol intake, hypertension, diabetes, smoking, marital status, education, and total calorie intake.

*< 1/month* < one serving size/month, *1/month–1/week* one serving size/month ≤  ~  < one serving size/week, *1–3/week* one serving size/week ≤  ~  < three serving sizes/week, *≥ 3/week* ≥ three serving sizes/week.

Supplementary tables present the risk of depressive symptoms according to each of oyster mushroom consumption and other mushrooms consumption. Oyster mushroom consumption ≥ 1/month had the lower risk for depressive symptoms than rare/never consumption in both men and women (Supplementary Table 1). This association was observed only in age subgroup with age ≥ 40 years (Supplementary Table 2). Analysis for other mushrooms also showed the similar patterns of relationship with those in analysis for oyster mushroom, despite statistical insignificance in some cases (Supplementary Tables 3 and 4).

## Discussion

In a longitudinal analysis for 87,822 Koreans with mean age of 39.6 ± 6.9 years, we showed that high mushrooms consumption was significantly associated with the decreased risk of depressive symptoms. This association was independent of potential confounding factors for depressive symptoms such as age, BMI, sex, alcohol intake, hypertension, diabetes, smoking, marital status, education, and total calorie intake. In particular, it is noted that these findings were reproduced in both gender subgroup analysis and age subgroup analysis. Our results suggest that high mushrooms consumption has the protective effect on depression.

There have been studies supporting the potential benefit of mushrooms consumption in alleviating depressive symptoms. Clinical trials found the favorable effect of a specific type of mushroom on depressive symptoms among small number of study subjects^17,18^. Cross-sectional analysis for 24,699 U.S. adults showed the lower odds ratio and 95% CI for depression in the middle tertile of comprehensive mushrooms consumption than those with lowest tertile^19^. However, results from clinical trials can’t be extrapolated into the effect of general mushrooms consumption among general population because they focused only on a specific type of mushroom among a small number of subjects. Additionally, cross-sectional study can’t allow for the determination of the cause-effect relationship between mushroom consumption and the risk of depression. In contrast, our longitudinal analysis showed that high mushrooms consumption was significantly associated with decreased risk of depressive symptoms in 87,822 participants. Therefore, our results may provide clearer evidence for the potential benefit of mushrooms consumption on depression.

Mushrooms contain bioactive components with health benefit, which can be explanations for our findings. It has been proposed that inflammation and oxidative stress are involved in the pathophysiology of depression^20,21^. Subjects with depression have higher levels of inflammatory immune activation, along with other immunological changes^22^. Meta-analyses have demonstrated that peoples suffering from depression have an increase in proinflammatory cytokines including tumor necrosis factor-α and interleukin-6^23^. It has been suggested that oxidative stress plays a significant role in the pathophysiology of numerous neuropsychiatric disorders including major depression^21^. Studies demonstrated that the levels of oxidative stress markers such as superoxide dismutase, malondialdehyde and nitrite were altered in depressive disorders^24,25^. Therefore, it is postulated that anti-inflammatory agents and anti-oxidants contribute to prevent depressive disorder. Mushrooms are rich in antioxidants and anti-inflammatory agents. Mushrooms may be effective in reducing oxidative stress as are a potent source of powerful antioxidants^15^. Additionally, anti-inflammatory effect of mushrooms may have a protective function on depressive symptoms. In the present study, a major type of mushrooms is oyster mushroom that is most commonly consumed in Koreans. Oyster mushroom is considered as a functional food due to its anti-inflammatory and immunomodulatory activity^26^. Thus, it is plausible that anti-inflammatory and anti-oxidative activity in mushrooms lead to the decreased risk of depressive symptoms.

In our subgroup analyses by gender and age, all of subgroups show the similar pattern of relationship between mushrooms consumption and the risk of depressive symptoms. However, the protective effect of mushrooms consumption on depressive symptoms seems to be more prominent in women and people ≥ age of 40. It is challenging to identify the correct mechanism for this finding. However, it is likely that the protective effect of mushrooms consumption functions more strongly in high risk group for depressive symptoms. It is known that women have the higher prevalence of depression than men. Additionally, our study shows the higher incidence of depressive symptoms in women than men, which may lead to the stronger statistical power in women. In terms of age subgroup analysis, a survey from USA presented that the percentage of adults who experienced depressive symptoms was higher among those aged 45–64, compared with those aged 30–44^27^. Therefore, the results from our subgroup analyses may be explained by difference in the risk of depression and statistical power from number of incidence among subgroups.

CES-D scale was developed in 1977 to identify individuals with high risk for depression^28^. CES-D scale provides cutoff score (16 or greater), in which Lewinsohn et al. reported the good sensitivity and specificity and high internal consistency in cutoff score ≥ 16^29^. Nevertheless, there has been ongoing debate for the optimal cutoff in defining depressive symptoms. Some studies have demonstrated that cutoff in 20, 22, and 25 are more accurate in identifying depressive symptom than classic cutoff in 16^30,31^. Therefore, we conducted cross-sectional analysis with cutoff of 20, 22, and 25 to see if change of cutoff affects the association between mushrooms consumption and depressive symptoms. Even after change of cutoff, we could see decreased association with depressive symptoms in individuals with mushrooms consumption ≥ 1 month, compared with rare/never consumption. This result suggests that the protective effect of mushrooms consumption on depressive symptoms may be maintained by change in cutoff of CES-D.

The merits of the present study are large number of study participants, survey for comprehensive mushrooms consumption and investigation for depressive symptoms through CES-D scale. These merits allowed us to quantify the risk of depressive symptoms according to mushrooms consumption in all participants and each subgroup.

Nonetheless, our study has several limitations.

First, the incidence of depressive symptoms was evaluated only by CES-D scale. Despite reasonability and reproducibility of CES-D scale in epidemiological setting, CES-D scale is not a gold standard in diagnosing depressive disorder. Thus, there is a concern for the underestimation or overestimation of depressive symptoms.

Second, survey for mushrooms consumption is performed for oyster mushroom and other mushrooms. Therefore, we are unable to specify the type of mushrooms consumed in our study participants. Although oyster mushroom is most commonly consumed in Koreans, other mushrooms can be consumed as a major type. Our results should be interpreted as a protective effect of general mushrooms consumption rather than specific type of mushrooms.

Third, because FFQ was based on self-administered questionnaire, there was a possibility of misclassification for type and frequency of consumed mushrooms.

Fourth, our results are derived from epidemiologic observation, which is unable to provide accurate mechanisms and explanations for the association between mushrooms consumption and the risk of depressive symptoms. Further studies should be done to identify the correct mechanism for the protective effect of mushrooms consumption against depressive symptoms.

In conclusion, our study indicated that increase in mushrooms consumption more than specific levels was associated with the decreased risk of depressive symptoms. Subgroup analyses identified that this association is similarly observed in both gender and age subgroups. However, women and participants ≥ age of 40 showed the more prominent association between increased mushrooms consumption and the decreased risk of depressive symptoms than men and participants < age of 40. These findings suggest that high consumption of mushrooms has the protective effect on depressive symptoms.

## Method

### Study participants and exclusion criteria

Relevant clinical and sociodemographic data were obtained from Kangbuk Samsung Health Study (KSHS). KSHS is a cohort study to investigate the medical data of Koreans who have received medical health check-up in Kangbuk Samsung Hospital. Korea’s Industrial Safety and Health law orders that all of Korean employees should receive medical health check-up annually or biennially. Ethics approvals for the study protocol and analysis of the data were obtained from the institutional review board (IRB) of Kangbuk Samsung Hospital (IRB No. KBSMC 2020-09-25). All procedures performed in studies involving human participants were in accordance with the ethical standards of the IRB of the Kangbuk Samsung Hospital and with the 1964 Helsinki declaration and its later amendments or comparable ethical standards. IRB of Kangbuk Samsung Hospital approved the exemption of informed consent for the study because we only assessed retrospective data with de-identified personal information obtained from routine health check-up.

**Figure 1:**
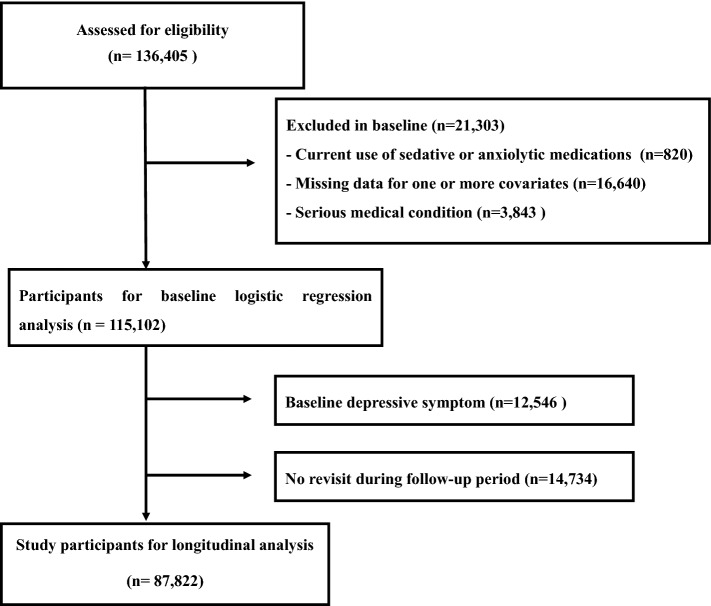
Flowchart of enrolled study participants.

Using data of KSHS, we initially enrolled 136,405 participants who had responded to semi-quantitative food frequency questionnaire (FFQ) including mushrooms consumption and CES-D between March 2011 and December 2012. Among initial 136,405 participants, we excluded 21,303 participants according to exclusion criteria as follows: 820 participants with taking sedative or anxiolytic, 16,640 participants with missing value in covariate data (e.g. BMI, hypertension, education), and 3843 participants with a history of serious medical diseases (e.g. coronary heart disease, stroke, and cancer). We identified remaining 115,102 participants after exclusion, and enrolled them into the baseline cross-sectional analysis for mushrooms consumption and depressive symptoms defined by multiple cutoffs of CES-D^16,20,22,25^. In addition, to conduct longitudinal analysis for the risk of depressive symptoms (defined by CES-D ≥ 16) during follow-up period, we further excluded 12,546 participants with baseline depressive symptoms (CES-D ≥ 16) and 14,734 subjects who did not return during the follow-up period from 115,102 participants. We finally recruited 87,822 participants who revisited and responded to the CES-D questionnaire from January 2013 to December 2018 (Fig. 1).

### Clinical and sociodemographic data

Study data include medical history assessed by self-administered questionnaire, anthropometric measurements and laboratory measurements. All study subjects were asked to respond to a health-related behavior questionnaire, which included the topics of alcohol consumption, smoking and exercise. Marriage, education, income, and occupation, which may influence psychological state and dietary patterns, were also included in the questionnaire. Hypertension was defined as a prior diagnosis of hypertension or having a measured BP ≥ 140/90 mmHg at initial and follow up examinations. Trained nurses measured BP on sitting position by automatic device (53000-E2, Welch Allyn, USA) three times after a 5 min rest with at least 30 s interval. Final BP levels were obtained as average of second and third BP measurements. The BMI was calculated by dividing weight (kg) by square of height (m^2^). DM was defined as one of following conditions; fasting glucose ≥ 126 mg/dL, hemoglobin A1 c (HbA1c) ≥ 6.5%, and a prior diagnosis of DM^32^.

Blood samples were collected after more than 12 h of fasting and were drawn from an antecubital vein. The fasting serum glucose was measured using the hexokinase method, and hemoglobin A1c (Hba1c) was measured using an immunoturbidimetric assay with a Cobra Integra 800 automatic analyzer (Roche Diagnostics, Basel, Switzerland). Serum uric acid levels were measured enzymatically using an automatic analyzer Advia 1650 Autoanalyzer, Bayer Diagnostics; Leverkusen, Germany).

### Assessment of FFQ data

We assessed the dietary intake of KSHS participants using the FFQ that was developed for the Korean genome epidemiologic study. The dietary data to design the FFQ were obtained from the Korea Health and Nutrition Examination Survey^33,34^. A detailed description of the FFQ^33^ and its validation in the Korean population has been described in a previous study^34^. The frequency of food consumption was composed of nine categories (e.g., mushrooms consumption was categorized never or rarely, once a month, two or three times a month, once or twice a week, three or four times a week, five or six times a week, one times a day, two times a day, and more than three times a day) and three serving sizes for each food. In mushrooms consumption, one serving size in mushrooms consumption was 30 g and the serving size was classified into 15 g, 30 g, and 45 g per day.

Participants answered their consumption of oyster mushroom and other mushrooms were categorized into five group according to mushroom consumption as follows: rare/never, < 1/month (< one serving size/month), 1/month–1/week (one serving size/month ≤  ~  < one serving size/week), 1–3/week (one serving size/week ≤  ~  < three serving sizes/week), ≥ 3/week (≥ three serving sizes/week). Total energy and nutrient intake was calculated by the Can-Pro 3.0 software developed by The Korean Nutrition Society^35^.

### Assessment of depressive symptoms

Depressive symptoms were assessed using the Korean versions of CES-D scale^36^. The CES-D is a self-report questionnaire designed to assess the current presence of depressive symptoms in the general population^28^. We used the 4-factors 20-items CES-D Scale with scores ranging from 0 to 3, with 0 indicating that the depressive symptoms were experienced rarely and 3 indicating that depressive symptoms were experienced most of the time in the past week (e.g. “I thought my life had been a failure.” 0 = seldom (not at all or less than 1 day), 1 = sometimes (1 ~ 2 days), 2 = often (3 ~ 4 days), 3 = almost always (5 ~ 7 days)). The presence of depressive symptoms was defied using a classic cutoff of CES-D score (16 or greater)^28^. In addition, we adopted additional cutoffs of CES-D score in 20, 22, and 25 as validated in previous studies^30,31^.

### Statistical analyses

The baseline parameters among groups of mushrooms consumption are presented as means ± standard deviation for continuous variables and as proportions for categorical variables. Main clinical characteristics and parameters among study groups were compared using ANOVA for continuous variables and chi-square test for categorical variables.

A Cox proportional hazards model was used to calculate the unadjusted and multivariable-adjusted hazard ratio (HR) and 95% confidence intervals (CI) for depressive symptoms (multivariable adjusted HR [95% CI]) in each study group. The models were adjusted for multiple covariates including age, BMI, sex, alcohol intake, hypertension, diabetes, smoking, marital status, education, and total calorie intake. The covariates of the multivariable model were selected for the presence of significant differences between groups or established risk factors for depression. The incidence cases, incidence density (incidence cases per 1000 person-years), person years of each group were calculated. Trend analysis conducted with median value of mushroom consumption. The proportional hazards assumption was confirmed by log–log plots and Schoenfeld residual test. To verify multicollinearity between variables, we analyzed Variance Inflation Factor (VIF), and it was confirmed that there were no variables with VIF greater than 10.

Subgroup analyses were conducted by age (< 40 or ≥ 40 years old) and gender. In interaction test, age had the significant interaction with mushrooms consumption (P for interaction < 0.001), whereas gender fail to significant interaction with mushrooms consumption (P for interaction = 0.144). However, considering gender difference in the prevalence of depressive symptoms, we conducted subgroup analyses for both age and gender.

In a baseline cross-sectional study enrolling 115,102 participants, logistic regression analysis was used to analyze the relationship between mushrooms consumption and depressive symptoms defined by multiple cutoffs of CES-D^16,20,22,25^. The goodness of fit for logistic regression model was evaluated by Hosmer–Lemeshow test.

All statistical analyses were performed using R 3.6.3 (R Foundation for Statistical Computing, Vienna, Austria), and a value of P < 0.05 (two-sided) was considered statistically significant in all analyses.

## Supplementary Information

Supplementary Tables.

## Data Availability

The data that support the findings of this study are available from Kangbuk Samsung Cohort Study, but restrictions apply to the availability of these data, which were used under license for the current study, and so are not publicly available. Data are however available from the authors upon reasonable request and with permission of Kangbuk Samsung Cohort Study.
