# Supplementary material for: The protective effect of mushroom consumption on depressive symptoms in Korean population
Source: Sci Rep. 2022 Dec 19;12:21914. doi: 10.1038/s41598-022-26549-5 (PMC9763420; doi:10.1038/s41598-022-26549-5)
Supplement: Supplementary file 1 — Supplementary Tables. [file 41598_2022_26549_MOESM1_ESM.docx]

**Supplementary Table 1.** Hazard Ratio (HR) and 95% confidence intervals (CI) for depressive symptoms (CESD ≥ 16) according to the oyster mushroom consumption

| **Characteristics** |  |  | **Serving** |  |  | **P for trend** |
| --- | --- | --- | --- | --- | --- | --- |
|  | **Rare/never** | **<1/month** | **1/month - 1/ week** | **1-3/week** | **≥ 3/week** |  |
| **All participants (n)** | 17,211 | 13,450 | 41,370 | 11,226 | 4,565 |  |
| Unadjusted HR | 1.00 (Reference) | 0.99 (0.94 – 1.05) | 0.95 (0.91 – 0.99) | 0.97 (0.92– 1.04) | 1.08 (0.998 – 1.18) | 0.888 |
| Adjusted HR | 1.00 (Reference) | 0.98 (0.93 - 1.04) | 0.92 (0.87 – 0.96) | 0.90 (0.85 – 0.96) | 0.94 (0.86 – 1.02) | <0.001 |
| Incidence case [n, (%)] | 2,576 (15.0%) | 2,010 (14.9%) | 5,943 (14.4%) | 1,656 (14.8%) | 740 (16.2%) |  |
| Incidence density | 29.6 | 29.4 | 28.2 | 28.9 | 32.2 |  |
| Person year | 86,973 | 68,252 | 210,866 | 57,234 | 22,956 |  |
| **Men (n)** | 12,796 | 9,100 | 26,167 | 6,108 | 1,941 |  |
| Unadjusted HR | 1.00 (Reference) | 0.96 (0.89 – 1.03) | 0.93 (0.88 – 0.98) | 0.97 (0.89 – 1.05) | 1.03 (0.90 – 1.17) | 0.241 |
| Adjusted HR | 1.00 (Reference) | 0.97 (0.90 - 1.04) | 0.92 (0.87 – 0.98) | 0.95 (0.87 – 1.03) | 0.96 (0.84 – 1.09) | 0.037 |
| Incidence case [n, (%)] | 1,758 (13.7%) | 1,214 (13.3%) | 3,388 (12.9%) | 824 (13.5%) | 273 (14.1%) |  |
| Incidence density | 26.8 | 25.8 | 25.0 | 26.1 | 27.7 |  |
| Person year | 65,557 | 47,055 | 135,597 | 31,616 | 9,866 |  |
| **Women (n)** | 4,415 | 4,350 | 15,203 | 5,118 | 2,624 |  |
| Unadjusted HR | 1.00 (Reference) | 0.98 (0.89 – 1.08) | 0.89 (0.82 – 0.96) | 0.84 (0.77 – 0.93) | 0.93 (0.83 – 1.04) | 0.002 |
| Adjusted HR | 1.00 (Reference) | 1.00 (0.91 – 1.10) | 0.90 (0.83 – 0.98) | 0.86 (0.78 – 0.95) | 0.93 (0.83 – 1.04) | 0.004 |
| Incidence case [n, (%)] | 818 (18.5%) | 796 (18.3%) | 2,555 (16.8%) | 832 (16.3%) | 467 (17.8%) |  |
| Incidence density | 38.2 | 37.6 | 33.9 | 32.5 | 35.7 |  |
| Person year | 21,416 | 21,197 | 75,269 | 25,617 | 13,089 |  |

Adjusting covariates: age, BMI, sex, alcohol intake, hypertension, diabetes, smoking, marital status, education, and total calorie intake (sex excluded in gender subgroup analysis)

<1/month: <one serving size/month, 1/month-1/week: one serving size/month ≤ ~ < one serving size/week, 1-3/week: one serving size/week ≤ ~ < three serving sizes/week, ≥ 3/week: ≥ three serving sizes/week

Adjusted HR: multivariate-adjusted hazard ratio

**Supplementary Table 2.** Hazard Ratio (HR) and 95% confidence intervals (CI) for depressive symptoms (CESD ≥ 16) according to the oyster mushroom consumption in subgroups stratified by age

| **Characteristics** |  |  | **Serving** |  |  | **P for trend** |
| --- | --- | --- | --- | --- | --- | --- |
|  | **Rare/never** | **<1/month** | **1/month - 1/ week** | **1-3/week** | **≥ 3/week** |  |
| **Age < 40 years old** | 10,265 | 7,573 | 23,195 | 6,195 | 2,447 |  |
| Unadjusted HR | 1.00 (Reference) | 1.04 (0.96 – 1.12) | 0.98 (0.92 – 1.04) | 1.02 (0.94– 1.10) | 1.12 (1.00 – 1.24) | 0.475 |
| Adjusted HR | 1.00 (Reference) | 1.03 (0.95 - 1.11) | 0.95 (0.89 – 1.01) | 0.95 (0.87 – 1.03) | 0.97 (0.87 – 1.09) | 0.080 |
| Incidence case [n, (%)] | 1,556 (15.2%) | 1,189 (15.7%) | 3,472 (15.0%) | 964 (15.6%) | 415 (17.0%) |  |
| Incidence density | 28.9 | 30.0 | 28.4 | 29.5 | 32.4 |  |
| Person year | 53,914 | 39,693 | 122,288 | 32,715 | 12,802 |  |
| **Age ≥ 40 years old** | 6,946 | 5,877 | 18,175 | 5,031 | 2,118 |  |
| Unadjusted HR | 1.00 (Reference) | 0.93 (0.85 – 1.02) | 0.90 (0.84 – 0.97) | 0.91 (0.83 – 1.01) | 1.04 (0.91 – 1.17) | 0.285 |
| Adjusted HR | 1.00 (Reference) | 0.92 (0.84 - 1.01) | 0.86 (0.80 – 0.93) | 0.83 (0.75 – 0.92) | 0.88 (0.77 – 0.999) | <0.001 |
| Incidence case [n, (%)] | 1,020 (14.7%) | 821 (14.0%) | 2,471 (13.6%) | 692 (13.8%) | 325 (15.3%) |  |
| Incidence density | 30.9 | 28.7 | 27.9 | 28.2 | 32.0 |  |
| Person year | 33,059 | 28,560 | 88,578 | 24,518 | 10,154 |  |

Adjusting covariates: age, BMI, sex, alcohol intake, hypertension, diabetes, smoking, marital status, education, and total calorie intake

<1/month: <one serving size/month, 1/month-1/week: one serving size/month ≤ ~ < one serving size/week, 1-3/week: one serving size/week ≤ ~ < three serving sizes/week, ≥ 3/week: ≥ three serving sizes/week

Adjusted HR: multivariate-adjusted hazard ratio

**Supplementary Table 3.** Hazard Ratio (HR) and 95% confidence intervals (CI) for depressive symptoms (CESD ≥ 16) according to the other mushrooms consumption

| **Characteristics** |  |  | **Serving** |  |  | **P for trend** |
| --- | --- | --- | --- | --- | --- | --- |
|  | **Rare/never** | **<1/month** | **1/month - 1/ week** | **1-3/week** | **≥ 3/week** |  |
| **All participants (n)** | 11,554 | 13,963 | 44,665 | 12,536 | 5,104 |  |
| Unadjusted HR | 1.00 (Reference) | 0.97 (0.91 – 1.04) | 0.91 (0.86 – 0.96) | 0.93 (0.87– 0.996) | 1.04 (0.96 – 1.13) | 0.230 |
| Adjusted HR | 1.00 (Reference) | 0.99 (0.93 - 1.06) | 0.91 (0.86 – 0.96) | 0.89 (0.83 – 0.95) | 0.93 (0.85 – 1.01) | <0.001 |
| Incidence case [n, (%)] | 1,776 (15.4%) | 2,108 (15.1%) | 6,388 (14.3%) | 1,834 (14.6%) | 819 (16.0%) |  |
| Incidence density | 30.7 | 29.9 | 28.0 | 28.7 | 32.0 |  |
| Person year | 57,942 | 70,483 | 228,403 | 63,875 | 25,577 |  |
| **Men (n)** | 7,768 | 9,473 | 29,489 | 7,081 | 2,301 |  |
| Unadjusted HR | 1.00 (Reference) | 0.95 (0.88 – 1.03) | 0.90 (0.84 – 0.96) | 0.95 (0.87 – 1.04) | 0.99 (0.88 – 1.12) | 0.172 |
| Adjusted HR | 1.00 (Reference) | 0.97 (0.89 - 1.05) | 0.90 (0.84 – 0.96) | 0.93 (0.85 – 1.01) | 0.93 (0.81 – 1.05) | 0.012 |
| Incidence case [n, (%)] | 1,086 (14.0%) | 1,278 (13.5%) | 3,813 (12.9%) | 960 (13.6%) | 320 (13.9%) |  |
| Incidence density | 27.5 | 26.3 | 24.9 | 26.3 | 27.3 |  |
| Person year | 39,522 | 48,643 | 153,284 | 36,540 | 11,704 |  |
| **Women (n)** | 3,786 | 4,490 | 15,176 | 5,455 | 2,803 |  |
| Unadjusted HR | 1.00 (Reference) | 1.01 (0.92 – 1.12) | 0.91 (0.84 – 0.99) | 0.85 (0.77 – 0.94) | 0.95 (0.85 – 1.07) | 0.004 |
| Adjusted HR | 1.00 (Reference) | 1.03 (0.93 – 1.14) | 0.92 (0.84 – 0.9997) | 0.86 (0.77 – 0.95) | 0.95 (0.84 – 1.06) | 0.004 |
| Incidence case [n, (%)] | 690 (18.2%) | 830 (18.5%) | 2,575 (17.0%) | 874 (16.0%) | 499 (17.8%) |  |
| Incidence density | 37.5 | 38.0 | 34.3 | 32.0 | 36.0 |  |
| Person year | 18,421 | 21,841 | 75,120 | 27,335 | 13,873 |  |

Adjusting covariates: age, BMI, sex, alcohol intake, hypertension, diabetes, smoking, marital status, education, and total calorie intake (sex excluded in gender subgroup analysis)

<1/month: <one serving size/month, 1/month-1/week: one serving size/month ≤ ~ < one serving size/week, 1-3/week: one serving size/week ≤ ~ < three serving sizes/week, ≥ 3/week: ≥ three serving sizes/week

Adjusted HR: multivariate-adjusted hazard ratio

**Supplementary Table 4.** Hazard Ratio (HR) and 95% confidence intervals (CI) for depressive symptoms (CESD ≥ 16) according to the other mushrooms consumption in subgroups stratified by age

| **Characteristics** |  |  | **Serving** |  |  | **P for trend** |
| --- | --- | --- | --- | --- | --- | --- |
|  | **Rare/never** | **<1/month** | **1/month - 1/ week** | **1-3/week** | **≥ 3/week** |  |
| **Age < 40 years old** | 6,514 | 7,531 | 25,496 | 7,222 | 2,912 |  |
| Unadjusted HR | 1.00 (Reference) | 1.08 (0.99 – 1.17) | 0.97 (0.91 – 1.04) | 0.99 (0.91 – 1.08) | 1.11 (1.00 – 1.24) | 0.962 |
| Adjusted HR | 1.00 (Reference) | 1.10 (1.01 - 1.19) | 0.97 (0.91 – 1.04) | 0.95 (0.87 – 1.04) | 1.01 (0.90 – 1.13) | 0.077 |
| Incidence case [n, (%)] | 986 (15.1%) | 1,221 (16.2%) | 3,807 (14.9%) | 1,095 (15.2%) | 487 (16.7%) |  |
| Incidence density | 28.9 | 31.1 | 28.2 | 28.8 | 32.3 |  |
| Person year | 34,077 | 39,286 | 134,887 | 38,068 | 15,093 |  |
| **Age ≥ 40 years old** | 5,040 | 6,432 | 19,169 | 5,314 | 2,192 |  |
| Unadjusted HR | 1.00 (Reference) | 0.86 (0.78 – 0.95) | 0.83 (0.77 – 0.90) | 0.86 (0.78 – 0.95) | 0.95 (0.84 – 1.08) | 0.057 |
| Adjusted HR | 1.00 (Reference) | 0.87 (0.79 – 0.95) | 0.82 (0.76 – 0.89) | 0.81 (0.73 – 0.89) | 0.83 (0.73 – 0.95) | <0.001 |
| Incidence case [n, (%)] | 790 (15.7%) | 887 (13.8%) | 2,581 (13.5%) | 739 (13.9%) | 332 (15.1%) |  |
| Incidence density | 33.1 | 28.4 | 27.6 | 28.6 | 31.7 |  |
| Person year | 23,865 | 31,197 | 93,516 | 25,807 | 10,484 |  |

Adjusting covariates: age, BMI, sex, alcohol intake, hypertension, diabetes, smoking, marital status, education, and total calorie intake

<1/month: <one serving size/month, 1/month-1/week: one serving size/month ≤ ~ < one serving size/week, 1-3/week: one serving size/week ≤ ~ < three serving sizes/week, ≥ 3/week: ≥ three serving sizes/week

Adjusted HR: multivariate-adjusted hazard ratio
